# Tic Douloureux Cured by Galvanism

**Published:** 1871-01

**Authors:** 


### TIC DOULOUREUX CURED BY GALVANISM.

An interesting case is reported from the practice of Dr. Wilks, of a patient who first began to suffer from neuralgic pains in the left side of the head. At first the pains were slight and transient, but they gradually became worse and more frequent, until they were now excruciating, and brought on by slight causes, such as blowing the nose, talking much, or eating. The nerve involved appeared to be the first division of the fifth. She had lost all her teeth on that side; she had never had rheumatic fever or ague, and was in other respects in good health. She was ordered three minims of tincture of aconite three times a day, but this produced no improvement. On February 1st she was ordered a drachm of chloride of ammonia every six hours. On February 7, being no better she was ordered half a drachm of the hydrated peroxide of iron and three grains of sulphate of quinine every four hours. On February 11 she was no better, and was ordered fifteen grains of bromide of potassium every four hours. On February 17 she was no better. All medicine was ordered to be omitted, and a continuous current of galvanism from twenty-five cells to be applied; after this she gradually improved. On February 28 she was much better. On March 18 she went out, saying she was quite well. (See *British Medical Journal*, July 9.) *Practitioner*, *Aug*. 1870.

